# Supplementary material for: Potential of Molecular Culture in Early Onset Neonatal Sepsis Diagnosis: A Proof of Principle Study
Source: Microorganisms. 2023 Apr 7;11(4):960. doi: 10.3390/microorganisms11040960 (PMC10145526; doi:10.3390/microorganisms11040960)
Supplement: Supplementary file 1 [file microorganisms-11-00960-s001.zip › microorganisms-2235348-supplementary.pdf]

Supplemental Tables

Supplemental Table S1 Maternal risk factors for early-onset sepsis

Maternal risk factors	Red flag
Maternal parenteral antibiotic treatment with a suspicion of maternal sepsis during labor within 24 hours before or after birth.	Yes
Suspected or confirmed infection in another baby in the case of a multiple pregnancy	Yes
Invasive group B streptococcal infection in a previous baby	No
maternal group B streptococcal colonisation, bacteriuria or infection in the current pregnancy	No
Prelabour rupture of membranes for more than 24 hours before the onset of labour in term born infants	No
Rupture of membranes for more than 18 hours before a preterm birth	No
Pre-term birth following spontaneous labour before 37 weeks' gestation	No
Intrapartum maternal fever >38°C (regardless of epidural analgesia), or chorioamnionitis (suspected or proven)	No

Supplemental Table S2 Clinical signs and symptoms of early-onset sepsis

Neonatal clinical symptoms	Red flag
Respiratory distress with onset more than four hours postpartum	Yes
Neonatal epileptic seizures	Yes
Need for mechanical ventilation	Yes
Signs of shock	Yes
Altered behaviour or responsiveness	No
Feeding difficulties (for example, feed refusal)	No
Apnoea and/or bradycardia	No
Signs of respiratory distress (including grunting, recession, tachypnoea)	No
Hypoxia (for example, central cyanosis or reduced oxygen saturation level)	No
Signs of neonatal encephalopathy	No
Need for cardiopulmonary resuscitation	No
Persistent pulmonary hypertension of newborns	No
Temperature abnormality (lower than 36°C or higher than 38°C) unexplained by environmental factors	No
Local signs of infection (for example, on the skin or eyes)	No
